# Attenuation of microvascular function in those with cardiovascular disease is similar in patients of Indian Asian and European descent

**DOI:** 10.1186/1471-2261-10-3

**Published:** 2010-01-15

**Authors:** William D Strain, Alun D Hughes, Jamil Mayet, Andrew R Wright, Jaspal Kooner, Nish Chaturvedi, Angela C Shore

**Affiliations:** 1 Institute of Biomedical and Clinical Science, Peninsula Medical School (Exeter), University of Exeter, UK; 2 International Centre for Circulatory Health, NHLI Division, Faculty of Medicine, Imperial College London & Imperial College Healthcare NHS Trust, UK

## Abstract

**Background:**

Indian Asians are at increased risk of cardiovascular death which does not appear to be explained by conventional risk factors. As microvascular disease is also more prevalent in Indian Asians, and as it is thought to play a role in the development of macrovascular disease, we decided to determine whether impaired microcirculation could contribute to this increased cardiovascular risk in Indian Asians.

**Methods:**

Forearm skin laser Doppler fluximetry in response to heating and ischaemia was assessed in 83 Europeans (41 with angiographically confirmed atherosclerotic coronary artery disease (CAD) and 42 from the general population) and 84 Indian Asians (41 with CAD). Explanations for differences in microvascular function were sought using multivariate analysis including conventional cardiovascular risk factors.

**Results:**

Compared to ethnically matched control populations both Europeans and Indian Asians with CAD had poorer microvascular responses to heating than those without (117(95% CI 105-131) vs. 142(130-162) arbitrary units, (au) for Europeans and 111(101-122) vs. 141(131-153)au for Indian Asians) and to ischaemia (44(38-50) vs. 57(49-67)au & 39(34-45) vs. 49(43-56)au respectively). These differences were not accounted for by conventional cardiovascular risk factors. There was no ethnic difference in the attenuation of microvascular function associated with CAD.

**Conclusion:**

Patients of European and Indian Asian descent with symptomatic CAD have poorer microvascular maximal tissue perfusion and reactive hyperaemia in the skin compared to ethnically matched asymptomatic control populations. Despite the increased cardiovascular risk in Indian Asians, the attenuation of microvascular function associated with CAD was equivalent in the ethic groups. This suggests that in Indian Asians microcirculation does not explain the increased susceptibility to CAD.

## Background

People of Indian Asian descent are at increased risk of cardiovascular disease[1,2]. This is only partly explained by the increased prevalence and susceptibility to insulin resistance and associated central obesity, hyperglycaemia and dyslipidaemia[3]. These risk factors are also predictors of microvascular dysfunction[4], which may account for the increased incidence of microvascular target organ damage reported in people of South Asian decent including microalbuminuria [5,6], diabetic retinopathy [7] and nephropathy [8].

Although an ethnic difference in brachial artery endothelial function has previously been reported[9], comparisons of microvascular function (< 80 μm in diameter) have not been explored. We cannot assume that abnormalities observed in the large vessels reflect those in the microcirculation, given the differences in structure, function and regulation of these vessels. Skin vessels are an easily accessible location to explore the microvasculature. They can be investigated non-invasively in large patient cohorts and mirror disturbances in other vascular beds. These measures have previously been correlated with, microalbuminuria[4], diabetes[10]and left ventricular hypertrophy[11]. Disturbances in skin microcirculatory reactive hyperaemia correlate positively with Framingham risk scores[12] in otherwise healthy volunteers.

We hypothesised that people with proven coronary artery disease would have attenuated microvascular function compared to those without and that Indian Asians would have poorer microvascular responses than Europeans for any given degree of coronary artery disease.

## Methods

Recruitment details have been published elsewhere[13]. In brief, the study population was recruited from two sources: the first consisted of consecutively recruited men with established clinical coronary artery disease, referred to cardiology clinics at St Mary's Hospital and Ealing Hospital in London, UK, with a minimum 50% stenosis in at least one coronary artery and evidence of atherosclerosis elsewhere in the coronary arteries confirmed angiographically (CAD +ve). A control population of men with no known coronary artery disease, matched to CAD+ve volunteers by ethnic group and 5 year age band, were drawn from a family practice register in North-West London that used both of the hospitals as secondary referral centres (CAD -ve). More than 97% of individuals are registered on such practice registers[14]. Maintenance of registers of patients with coronary artery disease is an incentivised requirement under the family practitioner contract, therefore those with symptomatic atherosclerotic disease can accurately be excluded. This was further cross-checked prior to enrolment, by a review of patient history and medication list to ensure no subjects with known coronary artery disease were included due to any lag between diagnosis and updating the primary care register.

St Mary's Hospital local research ethics committee approved the study and all participants gave written informed consent.

### Study investigations

All participants were invited to attend St Mary's Hospital for investigation. Baseline demographic, medical history and lifestyle data were collected. Measures of height, weight and central obesity were performed according to a standard protocol[3]. A resting electrocardiogram (ECG) was performed and sitting brachial systolic (SBP) and diastolic (DBP) blood pressures and heart rate were measured as the average of three readings using a validated automatic device (Omron 705CP, Omron Healthcare Europe B.V., Hoofddorp, the Netherlands). Fasting bloods were taken and analysed by the on-site laboratory, which is a member of the appropriate U.K. National Quality Assessment Scheme. Glucose and lipid levels were determined using the AU600 Olympus Diagnostic analyser (Olympus Diagnostic Systems, Eastleigh, UK) and insulin was determined by Abbott Axsym immunometric assay (Abbott Laboratories, Abbott Park, Illinois, USA). CRP was measured with a highly sensitive sandwich enzyme immunoassay, using rabbit anti-human CRP immunoglobulin as a catching and detecting antibody (Dako, Copenhagen, Denmark)

### Microvascular assessment

As the skin is a thermoregulatory organ, resting measurements exhibit a high degree of within-person variability and dynamic responses to stimuli are preferred. We measured, using established protocols[15], responses to heating and to ischaemia in arbitrary units (au) with laser Doppler fluximetry (DRT4, Moor Instruments, Axminster, UK) in an air conditioned room with the temperature fixed at 22°C.

### Response to heating (maximum hyperaemia)

A brass 0.7 cm^2 ^area heated disc was fixed on a flat region, not overlying any large vessels, on the left forearm. This was maintained at 42°C for 30 minutes. The maximum hyperaemic response was assessed by measuring skin vessel flux (perfusion) at eight equidistant points in the disk area and averaged. The intra-subject, intra-observer variability over a period of 3 months or less is 8.2 ± 2.0% within this study. This was measured in 12 individuals, three of whom had diabetes. The variability within a single subject over a 10-year period is 10%.

### Response to ischaemia (reactive hyperaemia)

A sphygmomanometer cuff was inflated to 30 mmHg above systolic blood pressure for three minutes. During occlusion, the flux generated by Brownian motion of the interstitial fluid and intracellular fluid at the heated site was recorded (biological zero). At three minutes the occlusion was released prompting a reactive increase in flow. In an unheated area of skin the peak value of this flow was recorded. The intra-subject reproducibility of the peak response is 16.2 ± 2.8% studied in 22 individuals (10 with confirmed atherosclerotic disease) on 2 occasions.

### Statistical analysis

Analyses were performed using STATA version 8 (Macintosh Version; Statacorp LP, USA). All normal data are presented as mean ± SD. Skewed data were log transformed and are presented as geometric means (95% confidence interval). Biological zero was subtracted from maximum hyperaemic response and reactive hyperaemia in keeping with common practice [16-18]. Statistical significance for categorical variables was calculated using the chi-squared test and ANOVA for continuous variables. In exploring independent determinants of microvascular responses, a measure of each conventional risk factor (blood pressure, lipid profile, obesity and insulin resistance) that maximally explained the variance(R^2^) was included in a multivariate model. Significance of contribution to this model was tested by the likelihood ratio statistic (LRS). A result was deemed significant if p < 0.05. In keeping with the recommendations of Cupples[19] and Rothman[20] the measured significance of the variables of interest is reported without adjustment for multiple testing.

## Results

### Clinical characteristics

Patients of Indian Asian descent were younger than their European counterparts (Table 1), however had the same duration of diagnosed cardiovascular disease(from time of initial presentation to cardiologist), implying a younger age at onset of symptoms. In both CAD +ve and CAD -ve populations, Indian Asians had lower weight (p < 0.0001) and systolic (p = 0.005), but not diastolic, blood pressure than their European comparators. The CAD +ve volunteers had higher insulin and HbA_1_c than the CAD -ve population, and Indian Asians had higher HbA_1_c than Europeans whether recruited from cardiology services or the general practice register. There was no ethnic difference in use of statin or antihypertensive/anti-anginal therapy.

**Table 1:** Baseline characteristics (mean ± SD except skewed data where geometric mean (95%CI) shown) stratified by enrollment group and ethnicity

	No known CAD		Established CAD		P by	
Variable	Europeans	Indian Asians	Europeans	Indian Asians	Ethnicity	CAD group
N	41	43	42	41		
Age (years)	61 ± 7	58 ± 9	67 ± 9	61 ± 8	0.0001	0.0004
Time since diagnosis (days)			65 ± 7	72 ± 9	0.08	
systolic BP (mmHg)	143 ± 21	135 ± 16	144 ± 19	133 ± 15	0.005	0.8
diastolic BP (mmHg)	85 ± 10	84 ± 9	81 ± 8	79 ± 77	0.2	0.003
Heart Rate (bpm)	63 ± 11	65 ± 11	61 ± 9	65 ± 11	0.1	0.5
Weight (kg)	84 ± 15	72 ± 13	86 ± 12	77 ± 10	< 0.0001	0.08
BMI (kg/m^2^)	28 ± 4	26 ± 4	28 ± 4	27 ± 3	0.0009	0.2
Waist circumference (cm)	100 ± 12	94 ± 10	103 ± 8	100 ± 9	0.004	0.005
Waist:hip ratio	0.99 ± 0.06	0.99 ± 0.10	0.98 ± 0.08	1.01 ± 0.05	0.3	0.8
Fasting insulin* (iu/dl)	41(33-50)	47(39-58)	54(45-64)	66(54-79)	0.06	0.002
Fasting glucose (mmol/l)*	5.5(5.2 - 5.8)	5.6(5.3-6.0)	5.9(5.5-6.3)	6.2(5.6-6.8)	0.3	0.03
HbA_1_c (%)*	5.7(5.5-5.9)	6.2(6.0-6.4)	5.9(5.7-6.2)	6.7(6.3-7.1)	< 0.0001	0.006
eGFR (MDRD equation)	73 ± 14	78 ± 12	64 ± 13	68 ± 16	0.1	0.0004
C-Reactive protein(mg/l)*	1.7(1.1-2.7)	1.6(1.1-2.3)	2.3(1.6-3.2)	1.3(1.0-1.8)	0.1	0.7
Total cholesterol (mmol/l)	4.7 ± 1.0	4.8 ± 0.8	4.1 ± 0.9	4.1 ± 1.0	0.99	< 0.0001
HDL cholesterol (mmol/l)	1.17(1.08-1.26)	1.19(1.10-1.28)	1.14(1.06-1.23)	1.04(0.96-1.12)	0.3	0.04
Cholesterol:HDL ratio*	4.0(3.6-4.3)	4.0(3.7-4.2)	3.5(3.3-3.8)	3.8(3.5-4.2)	0.4	0.06
LDL cholesterol (mmol/l)*	2.8(2.5-3.2)	2.8(2.6-3.0)	2.1(1.9-2.4)	2.1(1.9-2.3)	0.9	< 0.0001
Triglycerides (mmol/l)*	1.2(1.1-1.4)	1.2(1.1-1.4)	1.3(1.1-1.5)	1.4(1.2-1.7)	0.4	0.1
Statin therapy n (%)	12(29)	9(21)	36(86)	33(81)	0.3	< 0.0001
Diabetes n (%)	6(15)	10(23)	9(21)	15(37)	0.07	0.1
FHx heart disease n(%)	7(17)	11(25)	21(50)	14(34)	0.6	0.004
Smokers (Current/Ex/Never)	12/20/9	8/12/23	4/29/9	3/16/21	0.0005	0.2
Maximal hyperaemia (au)*	145(130-162)	141(131-153)	117(105-131)	111(101-122)	0.4	< 0.0001
Reactive hyperaemia (au)*	57(49-67)	49(43-56)	44(38-50)	39(34-45)	0.06	0.0007

* Log transformed data

p = significance between disease and control group and between each ethnic group two way analysis of variance (ANOVA), with test for interaction

No known CAD (Coronary artery disease) recruited from general practice register with no documented history of, or medications suggested of coronary artery disease.

Established CAD recruited with established symptomatic atherosclerotic coronary artery with at least one > 50% stenosis and evidence of other atherosclerotic disease on angiography

Those with clinically apparent coronary artery disease (CAD +ve) had lower maximum microvascular hyperaemia and reactive hyperaemia compared to the CAD -ve comparators in each ethnic group. There was a trend (p = 0.06) towards lower reactive hyperaemia in Indian Asians compared to Europeans. Otherwise there were no ethnic differences in microvascular function in either maximum hyperaemia or reactive hyperaemia.

In keeping with previous work using this technique[10,21], there was a negative association between maximum hyperaemia and HbA_1_c and insulin, such that better microvascular tissue perfusion was associated with lower HbA_1_c and lower fasting insulin level(data not shown). Reactive hyperaemia was negatively associated with waist hip ratio and HbA_1_c. There was a weak association between maximum hyperaemia and diastolic blood pressure, but not with any other measure of blood pressure. There was no association between age and either maximum hyperaemia or reactive hyperaemia in the population as a whole, either by ethnicity or by recruitment group, as has been previously demonstrated in other populations[10,21]. Neither an age-stratified analysis (data not shown) nor adjustment for differences in age and risk factors made any material difference to the difference between the CAD +ve and CAD -ve groups. The modest difference in reactive hyperaemia between the ethnic groups was accounted for by dysglycaemia (table 2), however multivariate adjustment for age and smoking history and measures of blood pressure, dyslipidaemia and dysglycaemia did little to attenuate the impairment in both maximal and reactive hyperaemia seen in the CAD +ve group compared to the CAD -ve controls of each ethnic group.

**Table 2:** Microvascular parameters stratified by coronary artery disease group in each ethnic group presented as geometric mean (95% CI) after adjustment for age, mean arterial blood pressure, total cholesterol, HbA_1_**c and smoking history**

	No known CAD		Established CAD		P by	
Variable	Europeans	Indian Asians	Europeans	Indian Asians	Ethnicity	CAD group
Maximum hyperaemia (au)	139(125-155)	146(131-163)	114(102-128)	114(101-127)	0.3	0.002
Reactive hyperaemia (au)	58(50-68)	48(41-56)	41(33-49)	43(37-51)	0.5	0.006

No known CAD (Coronary artery disease) recruited from general practice register with no documented history of, or medications suggested of coronary artery disease.

Established CAD recruited with established symptomatic atherosclerotic coronary artery with at least one > 50% stenosis and evidence of other atherosclerotic disease on angiography

p = significance between disease and control group and between each ethnic group; two-way analysis of variance (ANOVA), with test for interaction after adjustment (principle values log transformed)

## Discussion

We have demonstrated that patients presenting to cardiac services with symptomatic atherosclerotic coronary artery disease have systemic microvascular dysfunction detectable in the skin, independent of conventional cardiac risk factors. Contrary to our expectations, however, we did not detect an attenuation of microvascular function in those CAD +ve patients of Indian Asian origin compared to Europeans, despite increased measures of insulin resistance.

Evidence from population studies suggest Indian Asians present with diabetes at a younger age and have more macro and microvascular complications[8,22]. In keeping with this, subjects in our Indian Asian cohort were younger and had a trend towards a greater prevalence of diabetes. We demonstrated a trend towards poorer reactive hyperaemia in the Indian Asians compared to their European counterparts, however this is accounted for by differences in glycaemic control. Clearly this study cannot determine whether dysglycaemia represents the mechanism of the association or is simply a confounding variable of other pathways such as the presence of molecular or autonomic changes, that might also be ethnicity related. Our hypothesis that Indian Asians would have attenuated microvascular function compared to their European counterparts was based on the premise that microvascular dysfunction would precede the development of symptomatic atherosclerosis and continue to deteriorate as atherosclerotic burden increases.

Urinary albumin excretion rate independently predicts cardiovascular mortality in diabetes[23], hypertension [24], and the general (largely non-diabetic) population right into the normal range [25-27]. Of course, this does not mean the process of increasing urinary albumin excretion, nor other measures of microvascular function as reported here, are truly independent of atherosclerosis. If, for example, the process that leads to symptomatic atherosclerosis is related to the process resulting in microvascular dysfunction either due to shared risk factors, shared susceptibility to said risk factors or as part of the same aetiopathogenic process, AER and cardiovascular risk will mirror each other but be independent. This is supported by the lack of association between conventional risk factors of atherosclerosis and microvascular measures, despite the clear differences in populations with and without proven cardiovascular disease. The impact of the multifactorial complexity of this process cannot be explored in this cross-sectional study, but requires longitudinal follow-up of these patients.

The failure to demonstrate an ethnic difference, therefore, may be a result of a selection bias, by recruiting those with a demonstrable susceptibility (CAD +ve) or resistance (CAD -ve) to the pathogenic steps that lead to atherosclerosis, or a genuine lack of ethnic difference in microvascular function. Within the population we are able to detect a difference of approximately 0.23 SD of any given variable with 80% power at 5% significance. Although smaller differences may be present, the clinical relevance of such differences would be of questionable significance.

### Study limitations

This is a cross sectional study hence we cannot reliably draw conclusions on cause or effect. By consecutively recruiting CAD+ve patients then age and ethnicity matching control subjects, there is an age discrepancy between the ethnic groups, such that the Indian Asians are younger. Although there is only a weak associated between age and microvascular function within our populations, it is possible that this difference in age may contribute to the lack of difference. However an age-stratified analysis, demonstrating similar inter-ethnic regression coefficients for microvascular measures in each 5-year age stratum, makes this an unlikely explanation.

## Conclusion

Contrary to expectations, we did not detect an ethnic difference in microvascular function between Indian Asians and Europeans either from the general population or with confirmed coronary artery disease. There is, however, attenuated skin microvascular function in patients attending cardiology services with proven atherosclerotic disease compared to a general population sample independent of conventional cardiovascular risk factors such as blood pressure smoking, dyslipidaemia and dysglycaemia. Further exploration of the aetiopathogenic association between coronary artery disease and microvascular dysfunction may guide research into novel therapeutic interventions.

## Competing interests

The authors declare that they have no competing interests.

## Authors' contributions

WDS participated in the design and conduct of the study, performed the statistical analysis and drafted the manuscript. AH participated in the design of the study, advised on the analysis and helped draft the manuscript. JM participated in the design of the study and patient recruitment from cardiology services at St Mary's Hospital. JK participated in the design of the study and patient recruitment from cardiology services at Hammersmith Hospital. NC conceived the study, advised on the analysis and helped draft the manuscript. ACS participated in the design of the study, supervised all microvascular assessments, advised on the analysis and helped draft the manuscript.

All authors read and approved the final manuscript.

## Pre-publication history

The pre-publication history for this paper can be accessed here:

http://www.biomedcentral.com/1471-2261/10/3/prepub
